# Quality Improvement Initiative to Reduce Admissions for Nephrotic Syndrome Relapse in Pediatric Patients

**DOI:** 10.3389/fped.2019.00112

**Published:** 2019-03-29

**Authors:** Olga Charnaya, Sun-Young Ahn

**Affiliations:** ^1^Division of Nephrology, Children's National Health System, Washington, DC, United States; ^2^Department of Pediatrics, The George Washington University School of Medicine, Washington, DC, United States

**Keywords:** nephrotic syndrome, children, quality improvement, patient education, reduced admission

## Abstract

**Introduction:** Childhood nephrotic syndrome is frequently seen in pediatric nephrology practice and often requires patient hospitalization for management. Numerous complications of this disease can be managed in an outpatient setting if brought to the attention of the medical team in a timely manner. Outpatient management will reduce healthcare cost and improve patient safety. The goal of this quality improvement initiative was to reduce admissions for nephrotic syndrome relapse from 8 to <5 admissions at a single center in a 3-month period.

**Methods:** Fish-bone analysis was used to determine barriers to early recognition of relapse and successful outpatient care. Patient education about the disease process was identified as the primary barrier. A standardized approach to patient education as well as educational materials were developed. Champions were identified within each stakeholder group to train and disseminate the new process. Admission counts were compared from 3 years prior to implementation to 2 years post-implementation. Clinic visits for nephrotic syndrome were tallied as a balancing measure. Patients were surveyed in the outpatient clinics about whether they had ever received the education as a process measure.

**Results:** Admission counts were reduced and met goal for the first 3 quarters that were examined; however, the number of admissions went above target in the last quarter. Clinic visit numbers did not change over the study period. Process measure showed that 75–80% of families were provided with nephrotic syndrome education.

**Conclusion:** A standardized approach to patient and family education about idiopathic nephrotic syndrome can reduce admissions for management of relapse. This will reduce healthcare expenditure as well as improve patient safety.

## Introduction

Nephrotic syndrome is a clinical diagnosis characterized by the presence of severe edema, significant proteinuria, hypoalbuminemia, and hyperlipidemia. The incidence of childhood idiopathic nephrotic syndrome (INS) is estimated at 1.52–16/100,000 children per year (1, 2). Approximately 70% of children with minimal change disease (MCD) will experience a relapse within 24 months of diagnosis after completing a 3-month steroid induction (3). Each relapse is associated with development of edema and can be further complicated by acute kidney injury (AKI), serious infection, and thrombosis (4, 5). Overall, children who respond well to steroids have a favorable long-term prognosis but frequently relapsing children may require additional immunosuppression (6).

The need for hospitalization during the initial episodes and relapses for children with nephrotic syndrome varies based on severity of symptoms at presentation and institutional practices. Most commonly, initial presentation requires admission for management of edema, initiation of steroid therapy, and for patient/family teaching and education. Relapses, when detected early in patients who respond to steroids, can frequently be managed in an outpatient setting (6–8).

Hospital systems are prone to error and recent data show that medical errors are the third leading cause of death in the United States (9). Reducing the need for admission will help to protect patients from potential adverse events as well as help to reduce overall healthcare expenditure. Review of institutional practices and recent patient encounters identified numerous patients with relapse of INS presenting to the emergency room with severe edema without prior notification to the nephrology staff of the relapse. This late presentation necessitated admission for management of the severe edema and unnecessarily burdened the healthcare system.

This manuscript will describe a single-center quality improvement initiative to reduce admissions for children with nephrotic syndrome relapse from 8 to <5 admissions for every 3-month period.

## Methods

Children (age 1–18 years) with steroid-sensitive nephrotic syndrome treated by the division of pediatric nephrology at Children's National Health System from 2012 to 2016 were included in the analysis. Patients were identified using division billing data by ICD-9 and ICD-10 codes, and then confirmed by chart review. Children with steroid-resistant disease, secondary nephrotic syndrome or genetic disease were excluded because the causes for admission in this population are different. Children with treatment-resistant disease often require frequent hospitalizations for management of edema and secondary complications of nephrotic syndrome because there are no home-based therapies that are effective and therefore a quality improvement initiative aimed at early initiation of home-based therapies is unlikely to improve their care. As this project was undertaken as a quality improvement project, detailed demographic information was not collected.

**Figure 1:**
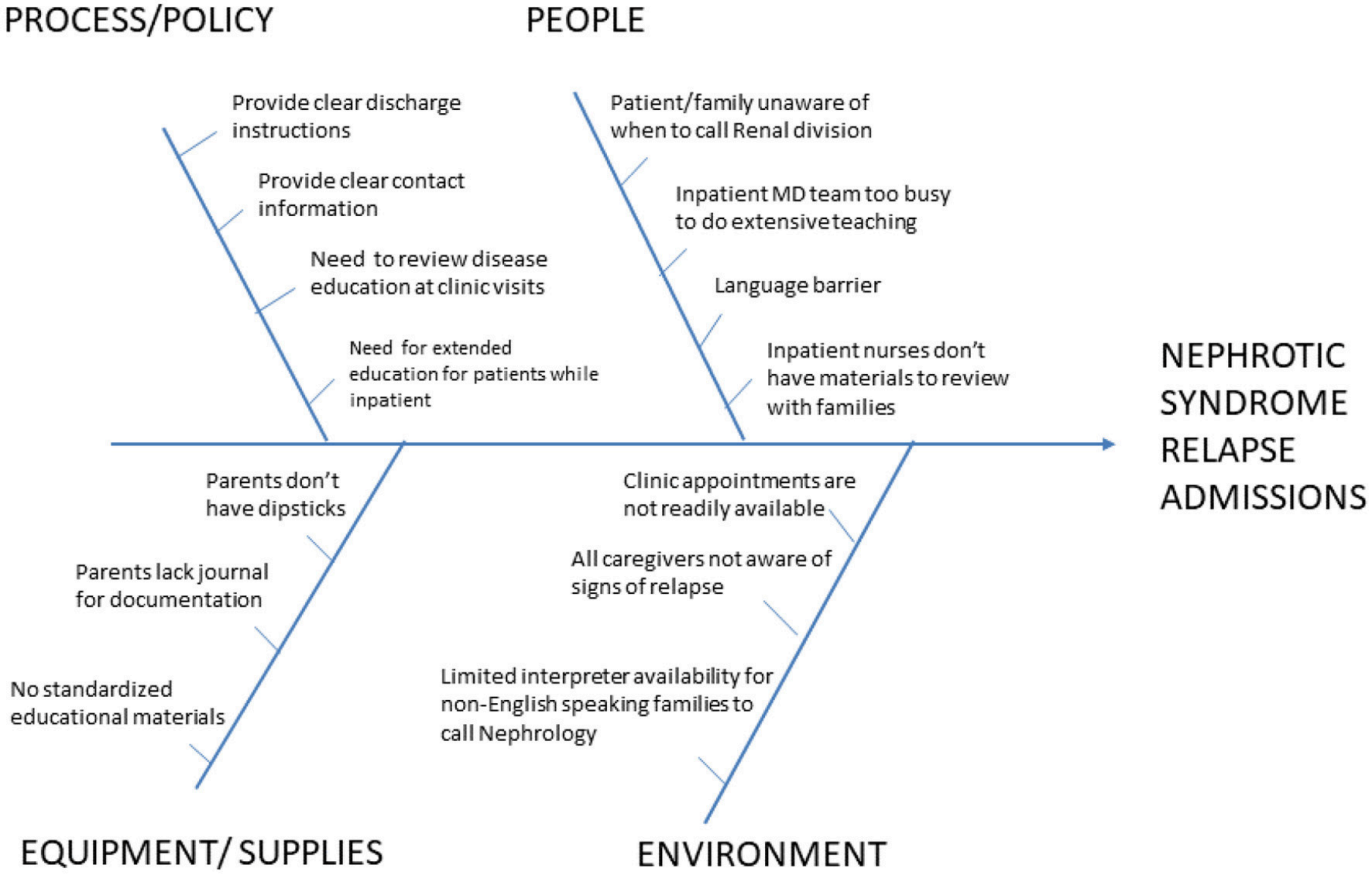
Fishbone analysis of factors contributing to admission for nephrotic syndrome relapse.

Fish-bone analysis was used to identify factors contributing to admissions specific to our institution and patient population (Figure 1). The primary factors chosen to be addressed were family education about the disease process, provision of urinary protein logging forms, easily available contact information and specific instructions for when to call the nephrology office. These elements were addressed in an educational booklet that was developed by the nephrology division (Supplementary Material). The booklet was given out to families at the time of diagnosis for new patients and at the time of clinic visit for returning patients. For new patients, this booklet served as an educational template that was reviewed with the family by the physicians and nurses on the team.

Baseline data was obtained by chart review for the 3 years prior to implementation of the education booklet. The outcome measure was number of admissions for each 3-month period observed. Observations were made during the fall and winter months, when higher volumes of nephrotic syndrome patients were seen due to concomitant viral infections. An assumption is made that there is a relatively stable patient population and data was tracked over numerous years to remove seasonal variation. The process measure involved random sampling of nephrotic syndrome patients in clinic to determine whether they had ever received a booklet. The balancing measure was the number of outpatient clinic visits for nephrotic syndrome during the same time-frame.

Three Plan-Do-Study-Act (PDSA) cycles were performed. The first cycle was an introduction of the educational booklet to newly diagnosed patients and their families by the project leader. This had a small scope but was received with positive feedback. The second cycle included revision of the handbook for readability by the hospital marketing team and engagement of all physicians in the pediatric nephrology division to distribute and review the booklet with nephrotic syndrome patients, both inpatient and outpatient. The third cycle included identification and engagement of inpatient nurse champions for the project. The nephrology floor charge nurses took the lead in distributing the booklet to all inpatients and reviewing the educational materials with them.

This is a project undertaken as a Quality Improvement Initiative at Children's National and it does not constitute human subjects research. As such it was not under the oversight of the Institutional Review Board. Statistical analysis was performed using Stata 15 (StataCorp. 2017. *Stata Statistical Software: Release 15*. College Station, TX: StataCorp LLC).

## Results

**Figure 2:**
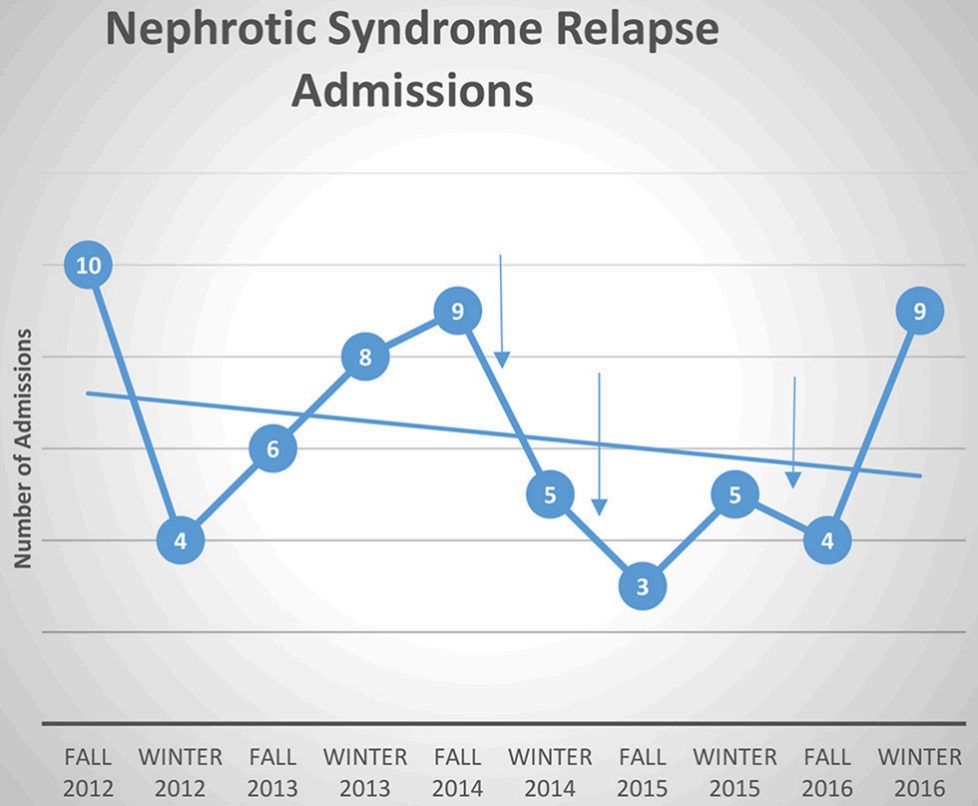
Admissions for nephrotic syndrome relapse over 5 years. Each arrow indicates time of PDSA cycle. Best fit line shows general trend toward decreasing number of admissions.

Of the available data, each 3-month cohort group included a range of 38–47 patients (inpatient + outpatient). There was no significant difference in the number of patients per cohort. The mean age of the patients was 7–10 years old (*p* = 0.01) with a slight male preponderance; gender distribution did not differ significantly between cohort groups (Table 1). Data on race or duration of diagnosis was not collected at the time of the project. Mean admission counts for the 3 years prior to implementation of the educational booklet in the fall and winter months was 7.4 admissions/3-month period. The average admission count after PDSA Cycle 1 was 5, PDSA Cycle 2 was 4, and after PDSA Cycle 3 was 6.5 admissions/3-month period (Figure 2).

**Table 1:** Demographic information for selected cohorts.

**Cohort**	**No. of patients**	**Mean age (years) [95% CI]**	**Male**
1	44	8.4 [7–9.8]	54.5%
3	38	7.4 [6.0–8.8]	55.3%
5	41	8.1 [6.7–9.5]	65.8%
7	41	10.8 [8.8–12.8]	61.0%
9	41	9.1 [7.7–10.5]	73.2%
10	47	10.9 [9–12.9]	66.0%
		*p* = 0.01^*^	*p* = 0.08

* *Statistically significant p < 0.05 obtained through ANOVA comparison of means*.

**Figure 3:**
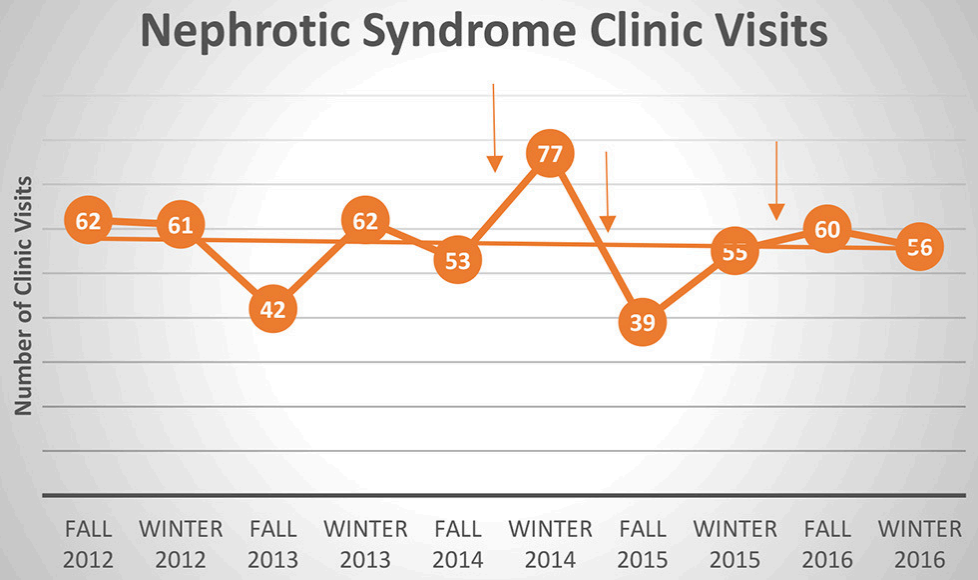
Clinic visits for nephrotic syndrome. Each arrow indicates time of PDSA cycle. Best fit line shows overall no change in clinic visits with each intervention.

Overall the clinic visit numbers for patients with nephrotic syndrome did not change. There was an average of 56 clinic visits/3-month period prior to implementation and an average of 57.4 clinics post-implementation (Figure 3). The process measure identified that 75–80% of families sampled reported having received an educational booklet.

An average inpatient hospital day is associated with $223 of professional services fees and $4,000 of facility fees. These vary based on the degree of patient acuity and complexity of care. An average hospital admission for relapse in a nephrotic syndrome patient lasted 3 days (rounded to whole day for billing purposes). Clinic visits are associated with an average of $189 of professional service fees; as no change in clinic visits was detected, there was not a net change in healthcare expenditure. However, a reduction in admission from 7.4 to 5 admissions per 3-month period correlates to an estimated healthcare cost reduction of $1,605 in professional fees and $28,800 in facility fees.

## Discussion

Childhood nephrotic syndrome has a variable course which often involves relapses. Relapses can be successfully treated in the outpatient setting, unless patients present late in their relapse course with significant symptoms. Insufficient family understanding about the disease process can lead to delay in coming to care and worse complications leading to the need for hospital admission. Standardization of family education reduced admissions for nephrotic syndrome relapse in our population.

Decrease in admissions was seen consistently except at the last time point. The failure to reach our goal at the last time interval may be as a result of the change made from physician to nursing-driven education and distribution of booklet. This may have resulted in a change in adherence to our intervention or different scope of education given to the families. Review of patients admitted in the last quarter also showed a higher proportion of families with poor medication adherence suggesting the presence of other psychosocial factors which may have contributed to delay in seeking care that would not have been addressed by our intervention. Finally, seasonal variation in the patient population and circulating viral illnesses could have contributed to the increased number of admissions. A major limitation of this quality improvement initiative is that data relies on the assumption of stable disease occurrence rates within our population.

Interestingly, there was no change in the balancing measure, which is the number of clinic visits. This suggests that patients were being managed remotely by phone calls/emails and not by direct face-to-face contact. While this is convenient for the patients, it may unnecessarily burden the healthcare providers (physicians and nurses) by increasing workload without being able to bill for their time. Data shows that physicians across specialties spend a significant amount of time with electronic medical record (EMR)/administrative tasks compared to direct patient care and that the burdens of excessive EMR documentation adds to physician burn-out (10, 11). Pediatric subspecialists were found to have the lowest pay, highest burden of medically and psychosocially complex patients, and most likely to experience symptoms of burn-out (12). Therefore, clinic visits may not be the most appropriate balancing measure and future quality initiative work should try to capture physician non-billable workload.

While this study was targeted at a patient population in a specific center, the challenges of managing children with nephrotic syndrome are similar across multiple geographic regions and patient populations. The specific education provided to families presented in this initiative is not center-specific and adheres to KDIGO (Kidney Disease: Improving Global Outcomes) recommendations, making the educational packet easily adaptable to use across multiple centers. There is no reported data, to our knowledge, of patient education for pediatric renal conditions resulting in reduced hospital admissions; however, there is data in other chronic disease conditions including pediatric asthma and adult chronic obstructive pulmonary disease (COPD). Patients who received self-management education were shown to have improved quality of life, reduced hospital admission, and emergency department visits (13, 14).

Overall reduction of admissions and outpatient management is beneficial for the patient due to less clinical complications, lower likelihood of being affected by medical error, and reduction of overall healthcare expenditure. Future quality improvement initiatives should aim to address institution specific determinants of hospitalization and should focus on assessing the degree of physician work-load. Current efforts include participation in a collaborative of 9 children's hospitals engaged in quality improvement efforts to improve vaccination rates and reduce admissions for children with nephrotic syndrome. Educational materials presented in this article are the basis for the multi-site intervention.

## Data Availability

The datasets generated for this study are available on request to the corresponding author.

## Author Contributions

OC contributed to the conception, data acquisition/analysis, and writing of the manuscript. S-YA contributed to the conception and writing of the manuscript.

### Conflict of Interest Statement

The authors declare that the research was conducted in the absence of any commercial or financial relationships that could be construed as a potential conflict of interest.
