# Supplementary material for: Quality Improvement Initiative to Reduce Admissions for Nephrotic Syndrome Relapse in Pediatric Patients
Source: Front Pediatr. 2019 Mar 29;7:112. doi: 10.3389/fped.2019.00112 (PMC6449647; doi:10.3389/fped.2019.00112)
Supplement: Supplementary file 1 [file Data_Sheet_1.PDF]

Nephrotic Syndrome

Family Education Booklet

Division of Pediatric Nephrology

This booklet is designed to provide you with an overview of your child's diagnosis and treatment. It will also prepare you for what to expect in the treatment of this disease.

Contents

The Kidney 3

Nephrotic Syndrome 3

Minimal Change Disease (MCD) 4

FSGS and Membranous Nephropathy 4

Biopsy 4

Checking for Protein 5

Dietary Changes 6

Complications 7

When to Call… 7

Calendar 8

Contact Information 12

Helpful Websites for More Information 12

The Kidney

Most people are born with two bean-shaped kidneys that grow with us through childhood. They are located in the middle of your back, one on each side.

The kidney's job is to remove toxins and extra water from our blood by making urine. The urinary track (ureters, urethra, bladder) take the waste out of the body when a person urinates (pees).

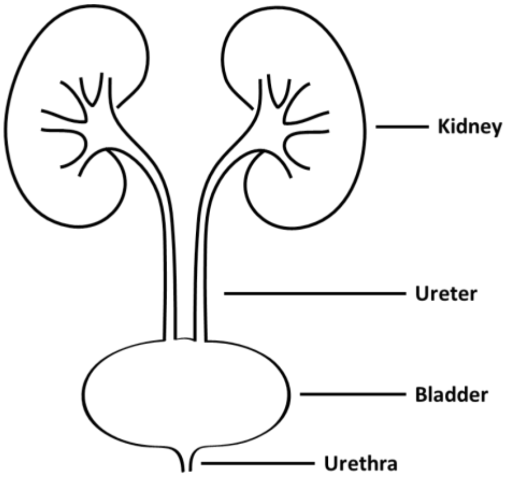

The kidneys also regulate many other body functions. This includes controlling your blood pressure by monitoring the amount of sodium (salt) and water that is wasted. They also regulate the electrolytes (sodium and potassium) and acid levels in the body.

Nephrotic Syndrome

Childhood nephrotic syndrome is not a disease by itself. It is a combination of symptoms and laboratory findings:

- Generalized swelling
- Low albumin level in the blood
- Protein in the urine
- Elevated cholesterol

All of these symptoms are a result of losing too much protein in the urine. The **diagnosis** of nephrotic syndrome is made by your doctor when they see this combination of symptoms and laboratory findings.

The swelling is most common around the eyes, in the legs and the scrotum in boys.
This happens because the job of the protein is to hold fluid in the blood vessels. When there are low protein levels in the blood, fluid leaks out into your other tissues and causes swelling.

The following conditions can cause nephrotic syndrome in children:

1. Minimal change disease
2. Focal segmental glomerulosclerosis (FSGS)
3. Membranous nephropathy

Common Causes

Minimal Change Disease (MCD)

This is the most common cause of nephrotic syndrome in children, comprising 90% of cases in children under the age of 10 years and more than 50% of cases in older children. The disease is so named because when biopsy samples are examined damage is not seen under the microscope. Scientists do not know what causes MCD but believe that it has something to do with the immune system.

The majority of children respond quickly to treatment and their swelling will improve dramatically. **Remission** occurs when the swelling is gone and they stop losing protein in the urine but most children will relapse. This often happens after the child gets sick with a cold or other illness. The rate of relapse varies for each child and may be as frequent as 4 times per year or as infrequent as once every other year. Each time they experience a relapse, they will be restarted on treatment and monitored closely. As children get older the relapses tend to become less frequent until they “grow out of” nephrotic syndrome by their teenage years. Children with **MCD** usually do not have any permanent kidney damage.

Focal Segmental Glomerulosclerosis (FSGS)

FSGS is the second most common cause of nephrotic syndrome in children. This disease typically does not respond well to initial treatment with steroids. It is diagnosed by biopsy. If a biopsy is necessary for your child, then the kidney doctor will speak to you about this possibility.

Membranous Nephropathy

Membranous nephropathy is very rare in children but is the most common cause of nephrotic syndrome in adults. This is also diagnosed by biopsy and will be discussed with you further if it is a possibility for your child.

Biopsy

A kidney biopsy is a procedure where a small piece of kidney tissue is removed with a needle. The procedure is done under anesthesia in an operating room.

The tissue is sent to a lab where it will be examined under a microscope. Other special tests are also done to better understand the causes of the kidney disease.

Children who have **minimal change disease** usually **do not need** a biopsy. A biopsy may be needed if any of the following occur:

- No improvement with treatment
- Frequent relapses
- A disease other than MCD is suspected

Treatment

Prednisone

The main treatment for nephrotic syndrome is a drug called **prednisone** (tablets) or **prednisolone** (liquid). This medicine is a steroid that helps to suppress the immune system and stop the attack on the kidney. Your child will be on a high dose of this medicine for 6 weeks and then weaned off slowly. Common side effects include:

- increased appetite
- weight gain
- irritability
- upset stomach
- difficulty sleeping

Diuretics

These are medicines that make your child pee a lot like **Lasix** or **Diuril.** The purpose is to remove some of the extra fluid that is making your child very swollen. A side effect of these medicines can be electrolyte imbalances, which we monitor closely.

Vaccines

Your child should not get certain vaccines while taking steroids. They should not get "live" vaccines such as FluMist, varicella (chicken pox) or measles-mumps-rubella (MMR). Most other vaccines are okay. Ask the doctor before your child gets any vaccines.

Checking for Protein

When you go home, you will have to check your child's urine once a day to monitor the change in protein lost. It is very important that you record this information and bring it with you to your follow-up appointment so that your doctor can review the information.

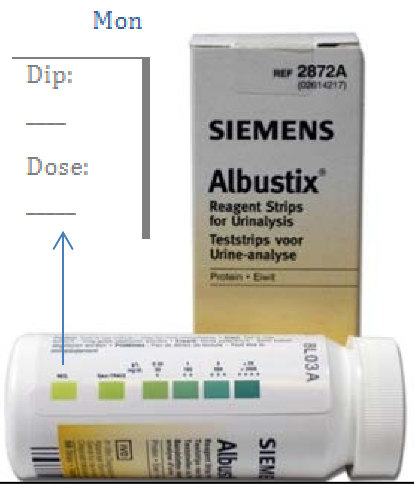

https://www.oncallmedicalsupplies.co.uk/albustix-x-50.html

To check the urine, have your child pee into a small cup. Dip the stick entirely into the urine. Line up the stick against the bottle and read results. Record results on your log.

- Do NOT dip the stick directly into the toilet.
- Do NOT use glucose sticks
- Use **only Albustix** to test for protein in the urine (albumin)

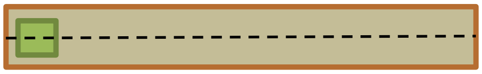

Some insurance companies do not pay for these sticks. They can be bought at most pharmacies or online on www.amazon.com. To make your supply of sticks last longer, you can cut them in half as shown by the dotted line in the diagram.

Dietary Changes

Since your child will be on steroid treatment, diet changes will be needed. Steroids can make children gain excess weight and retain salt, so maintaining a healthy diet is even more important than usual.

Low-Salt (Sodium) Diet

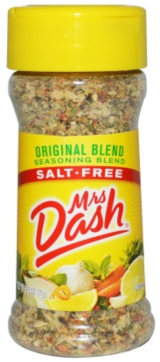

Limit your child's salt (sodium) intake to help reduce swelling and control blood pressure. Your child's appetite will increase and it is very important to choose low-salt and healthy snacks.

Tips for Maintaining a Low-Salt Diet:

- Do not add salt, soy sauce, or other seasonings that contain salt.
  (Mrs. Dash® is a great seasoning that has NO salt!)
- Serve foods in their natural state (avoid canned and processed foods)
- Use unsalted butter in cooking
- Read all labels to review the salt content of foods
- Fresh fruit, fresh vegetables, low-fat yogurt, and unsalted popcorn are good snack options.

Salt Limit: ___________

Fluid Restriction

Limit your child's fluids. The more fluids your child drinks will cause them to loose protein in the urine, which increases swelling. It is very important to maintain the fluid limit that is set.

Fluid Conversions and Common Fluid Volumes:

- 1 ounce = 30 mL
- 1 teaspoon = 5 mL
- 1 tablespoon = 15 mL
- 1 soda can = 12 ounces = 360 mL
- 1 standard glass = 8 ounces = 240 mL

Fluid Limit: _________

Families have found that it is easier to allow your child to drink small amounts of fluids throughout the day, as opposed to larger volumes of liquids less often.

Once your child has had negative or trace protein in their urine for 3 days in a row, the fluid restriction can be stopped.

A nutritionist will review more specific information with you before discharge. To schedule an outpatient appointment for further guidance, call: (202) 476-****

Complications

Infection

Children with nephrotic syndrome are at greater risk for infection. This is because some of the proteins being lost in the urine are antibodies, which are needed to fight infections. Additionally, steroids suppress the immune system.

Therefore, while undergoing the initial treatment course, young kids and everyone around them should wash their hands well. Keep them away from sick family members and friends.

Blood Clots

A blood clot can block the flow of blood and oxygen through a blood vessel anywhere in the body. Children with nephrotic syndrome are more prone to clots forming. Staying active can help prevent clots from forming. If a clot does form, your doctor will prescribe blood-thinning medicine.

High Cholesterol

As your child loses protein in the urine, her/his cholesterol level increases. The cholesterol levels return to normal quickly after the protein losses are stopped. If the cholesterol levels remain high, then your doctor may prescribe your child a medication to help lower the cholesterol.

When to Call…

You should call the nephrology office if any of the following happen while your child is on prednisone or prednisolone:

- Your child has fever (temperature greater than 100.4 F or 38.3 C measured under the arm or in the mouth)
- Your child complains of belly pain
- Your child was around anyone with the chickenpox

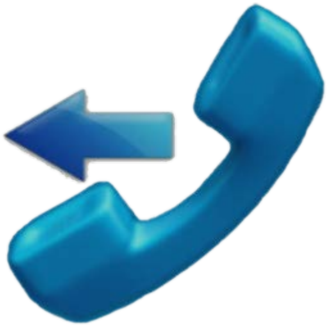

If your child is in remission and begins to have protein in the urine again, you should check the urine protein every day. Call the nephrology office if your child has 2+ or more protein for 3 days in a row. We will prescribe treatment over the phone and schedule a follow-up appointment. Do not wait until your child gets very swollen to call the office. If caught early, most relapses can be treated with medicines at home and do not require your child to be admitted to the hospital.

Calendar (Dip Stick Log)

Call our nurse when the dipstick is trace or negative for 3 days in a row.

Sun	Mon	Tue	Wed	Thu	Fri	Sat
Dip: ____ Dose: ____	Dip: ____ Dose: ____	Dip: ____ Dose: ____	Dip: ____ Dose: ____	Dip: ____ Dose: ____	Dip: ____ Dose: ____	Dip: ____ Dose: ____
Dip: ____ Dose: ____	Dip: ____ Dose: ____	Dip: ____ Dose: ____	Dip: ____ Dose: ____	Dip: ____ Dose: ____	Dip: ____ Dose: ____	Dip: ____ Dose: ____
Dip: ____ Dose: ____	Dip: ____ Dose: ____	Dip: ____ Dose: ____	Dip: ____ Dose: ____	Dip: ____ Dose: ____	Dip: ____ Dose: ____	Dip: ____ Dose: ____
Dip: ____ Dose: ____	Dip: ____ Dose: ____	Dip: ____ Dose: ____	Dip: ____ Dose: ____	Dip: ____ Dose: ____	Dip: ____ Dose: ____	Dip: ____ Dose: ____
Dip: ____ Dose: ____	Dip: ____ Dose: ____	Dip: ____ Dose: ____	Dip: ____ Dose: ____	Dip: ____ Dose: ____	Dip: ____ Dose: ____	Dip: ____ Dose: ____
Dip: ____ Dose: ____	Dip: ____ Dose: ____	Dip: ____ Dose: ____	Dip: ____ Dose: ____	Dip: ____ Dose: ____	Dip: ____ Dose: ____	Dip: ____ Dose: ____

Sun	Mon	Tue	Wed	Thu	Fri	Sat
Dip: ____<br>Dose: _____	Dip: ____<br>Dose: _____	Dip: ____<br>Dose: _____	Dip: ____<br>Dose: _____	Dip: ____<br>Dose: _____	Dip: ____<br>Dose: _____	Dip: ____<br>Dose: _____
Dip: ____<br>Dose: _____	Dip: ____<br>Dose: _____	Dip: ____<br>Dose: _____	Dip: ____<br>Dose: _____	Dip: ____<br>Dose: _____	Dip: ____<br>Dose: _____	Dip: ____<br>Dose: _____
Dip: ____<br>Dose: _____	Dip: ____<br>Dose: _____	Dip: ____<br>Dose: _____	Dip: ____<br>Dose: _____	Dip: ____<br>Dose: _____	Dip: ____<br>Dose: _____	Dip: ____<br>Dose: _____
Dip: ____<br>Dose: _____	Dip: ____<br>Dose: _____	Dip: ____<br>Dose: _____	Dip: ____<br>Dose: _____	Dip: ____<br>Dose: _____	Dip: ____<br>Dose: _____	Dip: ____<br>Dose: _____
Dip: ____<br>Dose: _____	Dip: ____<br>Dose: _____	Dip: ____<br>Dose: _____	Dip: ____<br>Dose: _____	Dip: ____<br>Dose: _____	Dip: ____<br>Dose: _____	Dip: ____<br>Dose: _____
Dip: ____<br>Dose: _____	Dip: ____<br>Dose: _____	Dip: ____<br>Dose: _____	Dip: ____<br>Dose: _____	Dip: ____<br>Dose: _____	Dip: ____<br>Dose: _____	Dip: ____<br>Dose: _____

Sun	Mon	Tue	Wed	Thu	Fri	Sat
Dip: ____<br>Dose: ____	Dip: ____<br>Dose: ____	Dip: ____<br>Dose: ____	Dip: ____<br>Dose: ____	Dip: ____<br>Dose: ____	Dip: ____<br>Dose: ____	Dip: ____<br>Dose: ____
Dip: ____<br>Dose: ____	Dip: ____<br>Dose: ____	Dip: ____<br>Dose: ____	Dip: ____<br>Dose: ____	Dip: ____<br>Dose: ____	Dip: ____<br>Dose: ____	Dip: ____<br>Dose: ____
Dip: ____<br>Dose: ____	Dip: ____<br>Dose: ____	Dip: ____<br>Dose: ____	Dip: ____<br>Dose: ____	Dip: ____<br>Dose: ____	Dip: ____<br>Dose: ____	Dip: ____<br>Dose: ____
Dip: ____<br>Dose: ____	Dip: ____<br>Dose: ____	Dip: ____<br>Dose: ____	Dip: ____<br>Dose: ____	Dip: ____<br>Dose: ____	Dip: ____<br>Dose: ____	Dip: ____<br>Dose: ____
Dip: ____<br>Dose: ____	Dip: ____<br>Dose: ____	Dip: ____<br>Dose: ____	Dip: ____<br>Dose: ____	Dip: ____<br>Dose: ____	Dip: ____<br>Dose: ____	Dip: ____<br>Dose: ____
Dip: ____<br>Dose: ____	Dip: ____<br>Dose: ____	Dip: ____<br>Dose: ____	Dip: ____<br>Dose: ____	Dip: ____<br>Dose: ____	Dip: ____<br>Dose: ____	Dip: ____<br>Dose: ____

My Questions for the Doctor…

Contact Information

Nephrology Office

111 Michigan Avenue
WW 1.5-100
Washington, DC 20010
Hours: 8:30am – 5pm
Phone: 202-***-****
Fax: 202-***-****

Nephrology RN Contact Information

Emergency On-Call Doctor: *********

Call after hours if you have an **URGENT** concern about your child's health
Ask for the nephrologist on-call and the operator will connect you

Helpful Websites for More Information

http://kidney.niddk.nih.gov/kudiseases/pubs/childkidneydiseases/nephrotic_syndrom/
http://www.kidneyfund.org/kidney-health/kidney-problems/childhood-nephrotic-syndrome.html
http://www.mayoclinic.org/diseases-conditions/nephrotic-syndrome/basics/causes/con-20033385

Works Cited

Gillespie, R. (2006, January). *KidneyWeb Pro*. Retrieved November 4, 2014, from Nephrotic Syndrome: http://kidneyweb.net/handouts.htm

Kidney Disease Basics. (2012, March 1). Retrieved Nov 4, 2014, from National Kidney Disease Education Program: http://nkdep.nih.gov/learn/kidney-disease-basics.shtml#what-your-kidneys-do
